# Live cell imaging at the Munich ion microbeam SNAKE – a status report

**DOI:** 10.1186/s13014-015-0350-7

**Published:** 2015-02-18

**Authors:** Guido A Drexler, Christian Siebenwirth, Sophie E Drexler, Stefanie Girst, Christoph Greubel, Günther Dollinger, Anna A Friedl

**Affiliations:** Department of Radiation Oncology, Ludwig-Maximilians-Universität München, Munich, Germany; Department of Radiation Oncology, Klinikum rechts der Isar, Technische Universität München, Munich, Germany; Institute for Applied Physics and Metrology, Universität der Bundeswehr München, Neubiberg, Germany

**Keywords:** Micro-irradiation, Particles, Live-cell imaging, Fluorescence microscopy

## Abstract

Ion microbeams are important tools in radiobiological research. Still, the worldwide number of ion microbeam facilities where biological experiments can be performed is limited. Even fewer facilities combine ion microirradiation with live-cell imaging to allow microscopic observation of cellular response reactions starting very fast after irradiation and continuing for many hours. At SNAKE, the ion microbeam facility at the Munich 14 MV tandem accelerator, a large variety of biological experiments are performed on a regular basis. Here, recent developments and ongoing research projects at the ion microbeam SNAKE are presented with specific emphasis on live-cell imaging experiments. An overview of the technical details of the setup is given, including examples of suitable biological samples. By ion beam focusing to submicrometer beam spot size and single ion detection it is possible to target subcellular structures with defined numbers of ions. Focusing of high numbers of ions to single spots allows studying the influence of high local damage density on recruitment of damage response proteins.

**Electronic supplementary material:**

The online version of this article (doi:10.1186/s13014-015-0350-7) contains supplementary material, which is available to authorized users.

## Introduction

Ion microbeams originally were designed to apply a defined number of ions to one or more cells in a cell population. In contrast to radiobiological experiments with a broad ion beam where the number of ion traversals of a cell varies over the population of cells due to Poisson distribution, application of defined numbers of ions minimizes cell-to-cell dose variation. In the beginning of biological research using ion microbeams, the main focus was placed on the impact of very small doses, down to one particle traversal within a whole cell population (reviewed in [1]). The possibility of targeting single cells with a defined number of ions, in combination with the huge progress of molecular and cellular biological techniques in the last two decades, opened a much broader field of biological/biomedical microbeam research (reviewed in [2]). Of special importance was the possibility of visualizing sites where DNA double-strand breaks (DSB) occurred by immunofluorescence staining of damage markers such as γ-H2AX [3].

**Figure 1:**
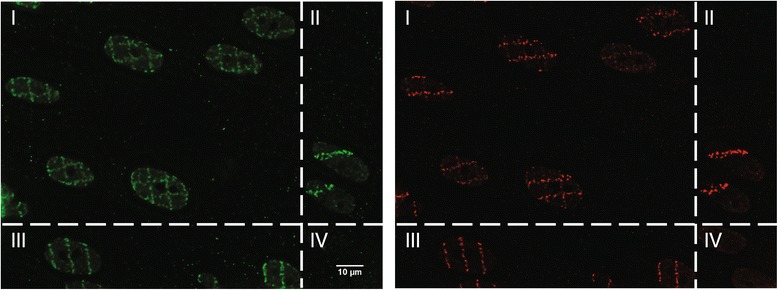
**Competition effect after sequential carbon ion irradiation at the Munich ion mirobeam SNAKE.** BJ1-hTERT cells (normal foreskin fibroblasts immortalized by ectopic expression of the catalytic subunit of the telomerase enzyme) were first irradiated in horizontal line pattern and after 45 min re-irradiated in vertical line pattern. The irradiation pattern is well reflected after immunofluorescence detection of γ-H2AX (green, quadrant II first irradiation only, quadrant III second irradiation only, quadrant I both irradiations, and quadrant IV no irradiation). In contrast, in cells irradiated twice 53BP1 foci (red) develop only at damage sites induced during the first irradiation. 53BP1 foci do not form in response to the second irradiation, although post-irradiation incubation was long enough to allow foci formation in cells that received only the second irradiation (compare quadrant I and III).

The ion microbeam SNAKE (Superconducting Nanoscope for Applied Nuclear Experiment), which is located at the Munich 14 MV tandem accelerator, originally was set up for applications in Materials Science [4]. Its main characteristic is a superconducting quadrupole lens for focusing the ion beam to a very narrow beam spot size [5,6]. In the year 2002, first steps were undertaken to modify SNAKE with the goal of performing radiobiological experiments. In the meantime, SNAKE has become one of a handful of ion microbeam facilities world-wide where biological experiments are routinely performed. A state-of-the-art microscope was integrated at the end of the beam line so that the focal planes of the beam and the microscope coincide at the position of the cell sample. This microscope, which also serves as the sample holder, allows microscopy with high optical resolution either with phase contrast imaging for positioning the cell sample or with fluorescence imaging to record fluorescent cellular structures of interest before and after irradiation. Initial experiments focused on irradiating cell monolayers with defined numbers of ions (mainly 55 MeV carbon and 100 MeV oxygen ions, see Table 1) applied in defined geometric patterns. Regions of several 100 μm in x and y direction (containing some tens of cells) were irradiated. After immunofluorescence detection of damage sites in fixed cells, the irradiation pattern is nicely reflected in emerging protein foci [6]. Thus, the location of radiation-induced DNA damage in the nucleus can be exactly located and sites of endogenous DNA damage can be excluded from analysis. This gains importance when spatially restricted and subtle alterations are investigated, such as radiation-induced modifications of epigenetic patterns [7]. Additionally, by choosing an intelligent pattern design, the effect of two sequential irradiations separated by an incubation interval (40 min up to several hours) during which cellular reactions to the first irradiation take place can be investigated since the irradiation pattern allows distinguishing between first and second irradiation (Figure 1). By sequential irradiation, it was discovered that foci formation of certain DNA damage response proteins (e.g. 53BP1, Rad51) is strongly reduced in cells that had been pre-irradiated (Figure 1), emphasizing the role of the binding and turnover characteristics of DNA repair proteins [8,9].

**Table 1:** **Ions used at SNAKE**

**Initial ion energy**	Ion energy at cell layer*	**LET at cell layer**	Dose per 1 ion traversal**
20 MeV p+	19.9 MeV	2.6 keV/μm	0.0022 Gy
55 MeV Carbon	42.9 MeV	368 keV/μm	0.30 Gy
100 MeV Oxygen	83.9 MeV	473 keV/μm	0.39 Gy

*Ion energy in live cell imaging setup, where cells are covered by 20 μm medium, calculated with TRIM/SRIM.

**Dose per nucleus was calculated assuming cylindrical nuclei of 200 μm^2^ area and 7 μm height.

One disadvantage of the analysis of radiation-induced redistribution of proteins and protein modifications via immunofluorescence reactions on fixed cells is that it is not suitable for the observation of very fast events or for detailed kinetics analyses. Therefore, a live-cell imaging (LCI) facility was installed at SNAKE [10]. By fusion of proteins which accumulate at DSB sites with fluorescent proteins [11] or use of chromobodies [12] the cellular response mechanisms to irradiation can be observed in real time. In the following sections, we present recent technical developments at the LCI facility and give examples of on-going research.

### Single ion detection for LCI experiments

Single ion detection ensures that each intended irradiation position is hit with exactly the desired number of ions. Ions accelerated at the Munich tandem accelerator have relatively low energies (see Table 1). To account for the limited ion ranges and the horizontal beam line, for conventional (i.e. not LCI) irradiation specific cell containers were constructed where cells grow on a thin Mylar foil. During irradiation (which takes a few minutes), cells are not submersed in medium, but a saturated atmosphere is generated by a second Mylar foil that serves as cover. With this setup, ion detection, which is necessary for counting of individual ions, takes place behind the sample [6] by using an ion detector that is integrated in the microscope at the position of an objective. In LCI experiments, however, cells are observed for prolonged times after irradiation, thus they have to be covered by sufficient medium to allow normal cell metabolism to take place. Special cell containers were designed in which the cells grow on a scintillator plate of 170 μm thickness that has optical properties very similar to cover glasses. Due to the optical quality of the scintillator plate high quality microscopy is possible. The single ion detection for 100 MeV oxygen and 55 MeV carbon ions in the live-cell imaging setup is performed by detection via the microscope objective of the light emitted when an ion hits the scintillator after traversing the sample. The detected light is guided within the microscope to a photomultiplier located at a camera port. The signal of the photomultiplier is used to control an electric deflection unit (chopper) and a scanning unit in a similar manner as in the conventional irradiation mode [10]. Shortly, the detector is connected to the chopper to shut down the ion beam after the desired number of ions was applied at one position of the sample. After shutting down the beam, the chopper triggers the beam scanning unit which moves the beam to the next spot to be irradiated [6]. Since scintillation signal and cellular fluorescence signal use the same light path, fast switching between irradiation mode and observation mode is possible.

For 20 MeV protons, single ion detection is performed as in the conventional setup, because the energy of a single particle is too low to cause light emission from the scintillator. Thus, after irradiation the photomultiplier has to be replaced by an objective and a gap of time of approximately 30 seconds between irradiation and image acquisition cannot be avoided.

During irradiation in the LCI mode, an approximately 20 μm thick medium layer is present between the beam exit nozzle and the cell layer [10]. This layer is thin enough to obtain a beam spot size of less than 0.5 μm full width half maximum. The targeting accuracy was measured to a standard deviation of 0.7 μm in x- and 0.8 μm in y-direction [13] which is dominated by uncertainties in the definition of beam and target position and by relative drifts between the optical microscope and the ion beam.

### Irradiation with defined patterns in LCI experiments

**Figure 2:**
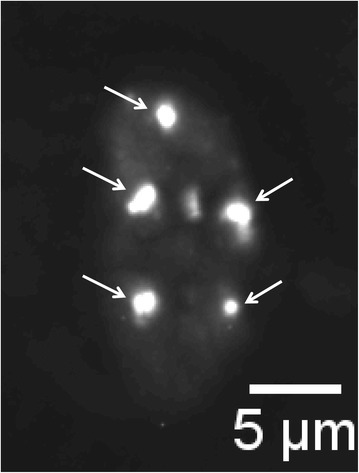
**Z-projection reveals irradiation pattern.** Hela cell stably transfected with a plasmid coding for a GFP- tagged version of the protein 53BP1 irradiated with 20 MeV protons (LET = 2.6 keV at cell position). 16 protons were applied per point in a 5 × 5 μm matrix pattern. Approximately 30 min after irradiation a series of images with defined z-distance through the whole nucleus was recorded (z-Stack). The images were deconvoluted to remove the blur caused by out-of- focus signals and a z-projection was performed. The radiation-induced 53BP1 foci are marked with arrows.

Even during live cell imaging where focus formation can be monitored in real time, the ability to irradiate cells with defined geometric patterns can be advantageous. By measuring the distances between DSB foci over time, we could show that damaged chromatin domains exhibit a mobility that is best described as sub-diffusion [14]. This may imply that adjacent DSB sites remain close to each other and long range movements are rare events, thus favoring correct rejoining of the DSB. Additionally, if several DSB occur in a cell, mis-rejoining and chromosomal aberration events are less likely. Irradiation in defined pattern is also helpful if only a few isolated DSB are induced by the particle traversal(s), e.g. by irradiation the cells with low linear energy transfer (LET) protons. In this case the radiation-induced foci do not necessarily appear in the same focal plane of the microscopic image. By using three-dimensional microscopy and recording images in z-direction through the nucleus, all the foci (radiation-induced and endogenously caused) are registered and after subsequent deconvolution of the z-stack, a z-projection can be performed to reveal the irradiation pattern (Figure 2). This helps to differentiate radiation-induced and spontaneous foci. In the example shown in Figure 2, the focused beam was used to apply 16 protons per point of the matrix, resulting in a local dose of 0.035 Gy. We found that at least 6–8 protons per point (i.e. 0.013 – 0.018 Gy) are necessary to obtain a discernible matrix pattern of the foci, i.e. roughly 1 DSB per irradiation point. This implies that about 56–77 DSB per Gy are produced under these conditions, a value that corresponds well to published data for DSB induction at low (0.1 Gy) dose [15].

### Locally concentrated high-dose irradiation

As shown above, by focusing the beam it is possible to place a high number of ions at a certain position. Recently it was shown that by focusing a high number of low LET protons to a submicrometer diameter, and thus changing the microdose distribution, enhanced biological effectiveness is generated [16].

**Figure 3:**
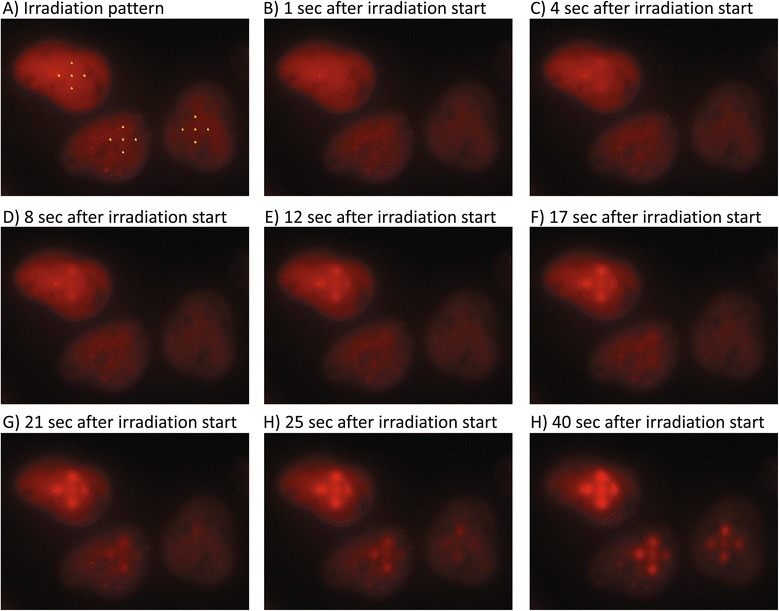
**Selective high-dose irradiation and PCNA recruitment. (A)** Target definition prior to irradiation in three cells selected from a whole microscopic field (see Additional file 1). Cells are to be irradiated with a five point cross with approximately 1000 carbon ions per point (each point was irradiated for 1 second with a particle fluence of about 1 kHz). For better illustration of the foci recruitment kinetics, indicated times refer to the start of the irradiation of the upper left cell, not to the irradiation start of the whole field of view. After the irradiation of the upper left cell, a cell not shown here was targeted (compare Additional file 1), followed by the lower left and the lower right cell. Thus irradiation of these cells took in total 20 seconds. Image acquisition started before irradiation. One second after irradiation start of the first cell (during irradiation of the second point in the first cell), no PCNA accumulation can be detected **(B)**. Three seconds later, while irradiation of the first cell is still in progress, already two sites of PCNA accumulation are clearly detectable **(C)**, Ongoing PCNA recruitment within the three nuclei **(D-H)**. See Figure 4 for a detailed analysis of the foci recruitment. Comparing the intensities of the foci of the upper left cell shows ongoing PCNA accumulation for at least 35 seconds **(C-H)**. PCNA protein is visualized using the “Cell Cycle Chromobody plasmid” (ChromoTek, Germany).

We reasoned that focusing of a large number of heavy ions to a submicrometer target and thus creating a high local damage density might facilitate detection of accumulation of those proteins that are recruited in low numbers to individual damage sites. Several proteins implicated in the response to DSB have been reported to accumulate at the sites of DNA damage after laser irradiation, while no accumulation could be visually detected after ionizing irradiation (e.g., [7,17,18]). One of the proteins described to accumulate after laser irradiation is PCNA [19]. Besides its function in various repair pathways, PCNA is involved in replication and accumulates at regions undergoing replication, which can be used to microscopically detect S-phase cells [20,21]. In preliminary experiments, we were not able to detect PCNA accumulation at damage sites induced by ionizing irradiation. To investigate whether the reason for the discrepancies observed with regard to PCNA and other proteins lies in the very high damage load induced by laser irradiation, we implemented a setup for irradiation of cells with up to several hundreds of ions per target point within a reasonable time span. The size of the region where the ions concentrate reflects the beam spot size (i.e. < 1 μm). In order to limit irradiation times, for this approach prior to irradiation individual cells are selected and the corresponding irradiation patterns are programmed for each target cell. Figure 3 demonstrates target definition and the rapid development (<3 s) of PCNA foci after irradiation of 3 cells with approximately 1000 carbon ions (corresponding to a local dose of about 300 Gy) per point in a 5-point cross pattern (see Additional file 1 for the whole image field during target definition and Additional file 2 for the entire movie). Thus, PCNA recruitment to damage sites is detectable at high local damage density. Because of the very fast recruitment kinetics, microscopy had to be performed while irradiation of the field was ongoing. Under these conditions, single ion detection is not possible. Rather, the number of ions applied to one point was averaged by the particle fluence and the time of irradiation.

**Figure 4:**
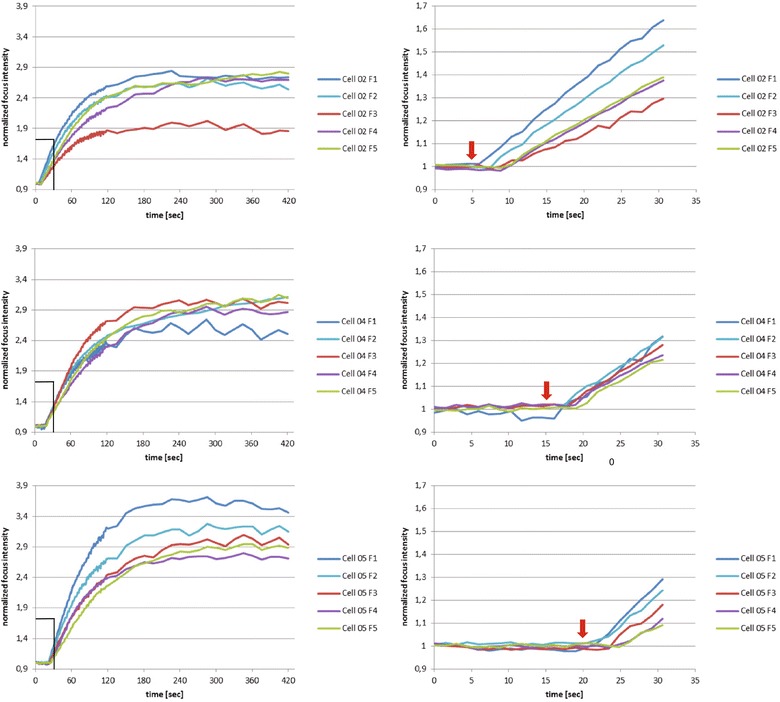
**Detailed kinetic analysis of the recruitment of PCNA in the cells shown in Figure** **3** **.** The left panels show for each cell and focus the recruitment of PCNA over the complete observation time span of 420 seconds. Note that 0 seconds (start of the irradiation of the whole field shown in Additional files 1 and 2) refers to a cell not shown in Figure 3. Numbering of foci reflects the sequence of irradiation upper (F1)-lower (F2)- left (F3)-right (F4)-center (F5). Irradiation of each point took about 1 s. The right panels show a magnification of the first 30 seconds of the observation. The irradiation start of each individual cell is marked by a red arrow.

Figure 4 shows the recruitment kinetics for each cell and the individual foci shown in Figure 3. It is evident that recruitment velocity and signal intensity differ between foci. A detailed investigation of the factors influencing recruitment characteristics is in progress.

Of interest, in these experiments we visualized endogenous PCNA by stable transfection of cells with “Cell Cycle Chromobody plasmid” (ChromoTek, Germany). This plasmid encodes a small fluorescence-tagged antibody (a so-called chromobody) against PCNA, which is derived from heavy chain antibodies of camelids genetically fused to a fluorescent protein [12,22]. Thus the endogenous PCNA protein can be monitored without the need for overexpression of PCNA fusion proteins, thus reducing the risk of artifacts and negative effects of overexpression.

### Targeted irradiation of cellular substructures

**Figure 5:**
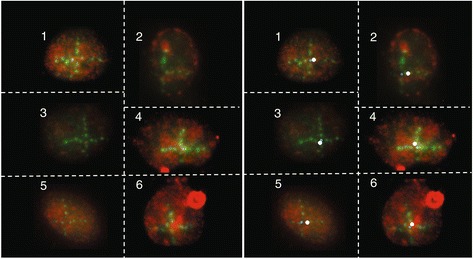
**Targeted irradiation of fluorescence labeled chromatin domains.** A TexasRed labeled nucleotide analogue was introduced via electroporation into Hela cells already stably transfected with a plasmid coding for a MDC1-GFP fusion protein (green). When present during S-phase of the cell cycle, the labeled nucleotide is incorporated into the DNA. After 72 h (2–3 cell divisions) separate chromatin domains (red) can be detected. One of these was chosen to be irradiated with a single carbon ion as the center of a nine point cross like pattern. The figure shows MDC1-GFP accumulation one minute after irradiation at the sites of DNA damage, which reflects nicely the irradiation pattern and gives the possibility to check if targeting was successful. In the left panel of the figure the irradiated cells are shown, in the right panel additionally the intended target is shown as white dot and the actual center of the irradiated cross-wise pattern is indicated by the blue dot. Note that we explain the strong red staining at the rim of cell 6 by conglomeration of TexasRed or TexasRed-dUTP. The ring-like structure suggests that the signal is located out-of-focus of the microscopic image, i.e. above or below the cell.

The combination of a very precise beam spot and the ability to perform targeted irradiation makes it possible to selectively irradiate defined subcellular targets. Of course, since the ions traverse the cell, targeting can only be accomplished in x-y direction, but not in z. This means that any object that lies in front of or behind the target will also be irradiated. Due to the radial dose distribution of high LET ions, in our case mostly carbon ions (LET = 368 keV at the cell layer), more than 90% of the DSB occur within a radial distance of 0.1 μm from the core of the ion track, and at a radial distance of 0.8 μm the dose practically drops to 0 Gy [16,23]. Thus, for larger targets, such as nucleoli, dose in the target depends mainly on targeting accuracy. Nucleoli have a diameter of approximately 3 μm. The height of nucleoli in fixed samples of U2OS cells is on average 2/3 of the nucleus height. Until now, it is an open question which role nucleoli play in radiosensitivity. To address this and similar questions, SNAKE was equipped with a self-developed semi-automated target recognition software. The software recognizes the correct targets (in this case nucleoli) in 70 – 100% of the cases, which requires manual correction of automatically determined targets. The probability for hitting the chosen nucleoli targets was then higher than 80% [13]. For smaller targets higher targeting accuracy is required. One example are fluorescence-labeled chromatin domains generated by incorporation of fluorescent nucleotides and subsequent cell growth for several generations. These domains are approximately 0.5 μm in size, which is close to the dimension of the achieved beam spot size. Thus, targeting of these small structures up to now has only partially been successful (see Figure 5).

### Long-term live-cell imaging

Live-cell imaging for several hours (up to 16 h) after irradiation at SNAKE has been described [10,14,24]. During these experiments, microscopy of the irradiated cell sample was conducted at the beamline. As a consequence, the beamline was blocked for the time of observation. Thus, we decided to transfer samples for long-term observation in the range of 24 h or more to a second microscope that is equipped similar to the beamline microscope. To easily relocate the irradiated position in the sample, the scintillator on which the adherent cells grow, contains marks with which a relative position of the irradiation site can be determined. In first experiments with non-irradiated cells we were able to track a cell undergoing a complete cell cycle by fluorescence microscopy for more than 50 h, without any indications of phototoxicity (Additional file 3).

### Conclusions and outlook

The live cell imaging facility at SNAKE is now able to routinely perform ambitious experiments. In addition to recording time series after single ion irradiation in defined patterns [24], experiments with selective high dose irradiation of cells within a reasonable time span can be performed. This raises the possibility to define the minimal dose needed for DNA repair proteins to accumulate in visible foci. Further, cellular substructures like nucleoli or different chromatin states (heterochromatin vs. euchromatin) can be targeted and irradiated with a defined number of ions, e.g. to investigate differential response mechanisms. At the moment, the setup is modified to perform three-color LCI experiments. This will enable us to either study the behavior of three different proteins tagged with different fluorescent markers or to include the cell cycle state of the targeted cells into the study.

At present, experiments at SNAKE mainly aim at elucidating basic mechanisms, but we do see a potential for more clinically oriented research, e.g. in testing compounds. In addition, SNAKE has proven invaluable for testing the effects of ultra-high dose rates that are expected to occur in tumor irradiation with laser-driven ions [25-31].

## Additional files

Additional file 1:
**Defined targets and order of irradiation.** U2OS cells stably transfected with the “Cell Cycle Chromobody plasmid” (ChromoTek, Germany) were marked for targeted irradiation with approximately 1000 carbon ions per point in five point cross-like pattern (artificial red dots). Since the irradiation per point takes one second, the order of irradiation is registered (red numbers). The irradiation order of the spots of a five point cross is upper-lower-left-right-center. The image is one step in the workflow for the acquiring of a time lapse series after targeted irradiation (Additional file 2). Additional file 2:
**Time lapse movie after targeted irradiation of U2OS cells with visualization of PCNA protein accumulation at sites of DNA damage.** The cells shown in Additional file 1 were irradiated with approximately 1000 carbon ion per point, which takes about 1 s per point. Irradiation of the first point started at time 00:00:00. Over time, PCNA protein accumulates at the sites of carbon ion induced DNA damage. Additional file 3:
**Long term fluorescence microscopy.** Fluorescence microscopy of U2OS cells stably transfected with the “Cell Cycle Chromobody plasmid” (ChromoTek, Germany) was performed for a total time of 59 h 45 min. Every 15 min an image was acquired and mounted to a movie (relative time of each image is shown). One cell (marked with an arrow) could be traced for more than 50 h, throughout a whole cell cycle. Starting from late S-phase (0 min until 4 h 05 min, as indicated by the PCNA staining pattern), it goes then through G2-phase (4 h 05 min until 12 h 45 min). First signs of mitosis can be seen at 13 h 00 min, and 17 h 15 min after starting the follow-up cell division seems to be completed and 2 daughter cells in G1-phase appear (until 19 h 29 min). At 19 h 45 min the lower daughter cell enters S-phase, while the upper one starts S-phase 2 h later at 21 h 45 min. The lower daughter cell then disappears out of the field of view. For the remaining cell, early S-phase takes 14 hours 30 min, followed by 9 hours mid S-phase and 2 hours late S-phase before entering G2-phase again.

## Abbreviations

53BP1: p53 binding protein 1
DSB: DNA double-strand break
FWHM: Full width half maximum
GFP: Green fluorescent protein
Gy: Gray
HR: Homologous recombination
LET: Linear energy transfer
MDC1: Mediator of DNA damage checkpoint 1
NHEJ: Non homologous end joining
SD: Standard deviation
SNAKE: Superconducting nanoscope for applied nuclear (Kern) physics experiments
