# Supplementary figures and images for: Live cell imaging at the Munich ion microbeam SNAKE – a status report
Source: Radiat Oncol. 2015 Feb 18;10:42. doi: 10.1186/s13014-015-0350-7 (PMC4341815; doi:10.1186/s13014-015-0350-7)

## Slide 1
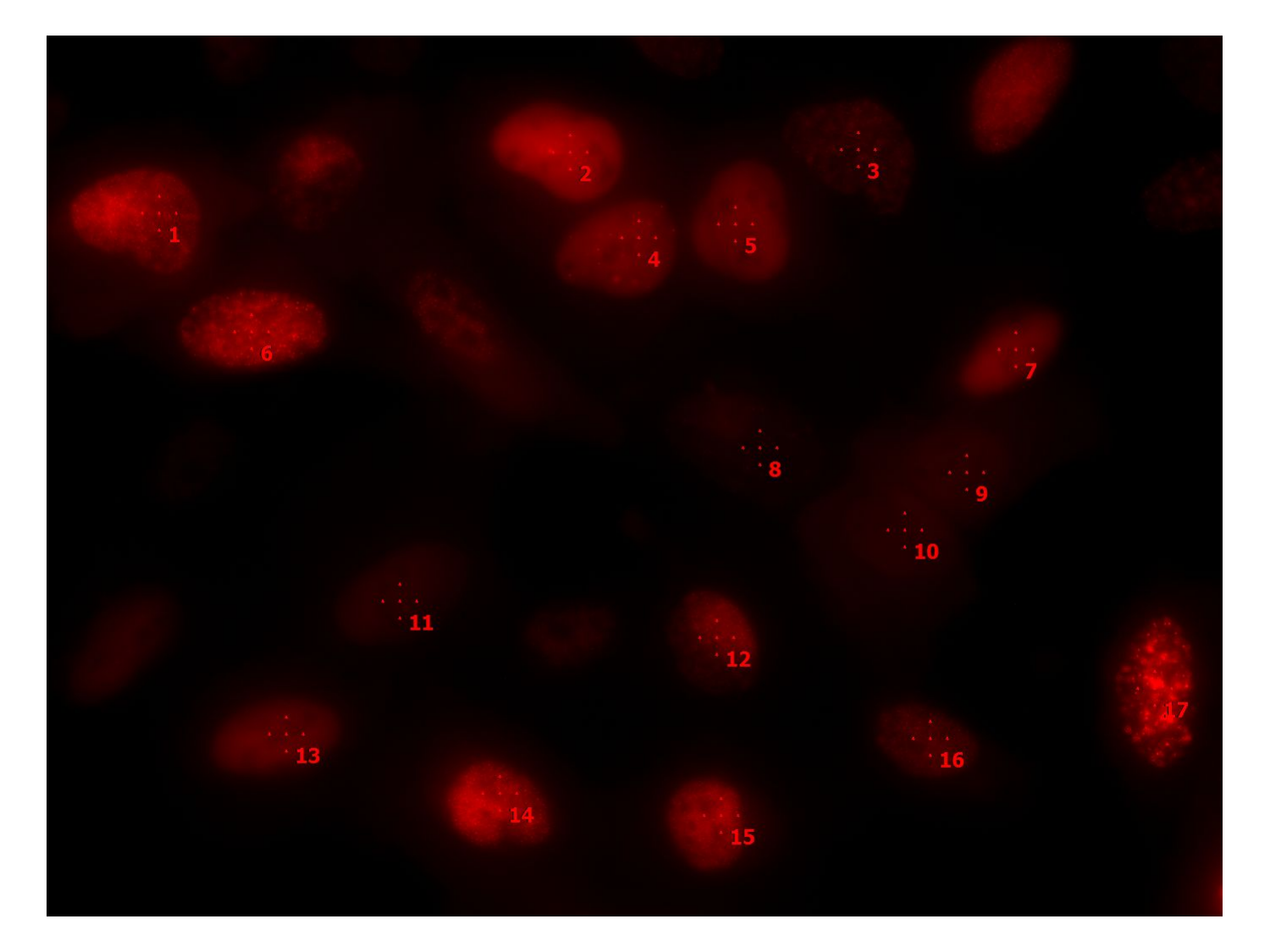

Supplement: Additional file 1: — Defined targets and order of irradiation. U2OS cells stably transfected with the “Cell Cycle Chromobody plasmid” (ChromoTek, Germany) were marked for targeted irradiation with approximately 1000 carbon ions per point in five point cross-like pattern (artificial red dots). Since the irradiation per point takes one second, the order of irradiation is registered (red numbers). The irradiation order of the spots of a five point cross is upper-lower-left-right-center. The image is one step in the workflow for the acquiring of a time lapse series after targeted irradiation (Additional file 2). [file 13014_2015_350_MOESM1_ESM.pptx]
